# Sex differences in one-year recurrence and all-cause mortality following catheter ablation of ventricular tachycardia in structural heart disease

**DOI:** 10.3389/fcvm.2022.1061471

**Published:** 2022-12-06

**Authors:** Patrik Tóth, Ferenc Komlósi, Péter Vámosi, Bence Arnóth, Nándor Szegedi, Zoltán Salló, Katalin Piros, Péter Perge, István Osztheimer, Pál Ábrahám, Gábor Széplaki, Béla Merkely, László Gellér, Klaudia Vivien Nagy

**Affiliations:** ^1^Heart and Vascular Center, Semmelweis University, Budapest, Hungary; ^2^Faculty of Medicine, Semmelweis University, Budapest, Hungary; ^3^Mater Private Hospital, Dublin, Ireland; ^4^Royal College of Surgeons in Ireland, Dublin, Ireland

**Keywords:** sex differences, ventricular tachycardia, catheter ablation, mortality, recurrence, women, structural heart disease

## Abstract

**Aims:**

We aimed to establish sex-specific predictors for 1-year VT recurrence and 1-year all-cause mortality in patients with structural heart disease undergoing catheter ablation.

**Methods:**

We analyzed data of 299 patients recorded in our structured registry. These included medical history, echocardiography parameters, laboratory results, VT properties, procedural data.

**Results:**

Out of the 299 patients, 34 (11%) were female. No significant difference was found between women and men in terms of VT recurrence (*p* = 0.74) or mortality (*p* = 0.07). In females, severe mitral regurgitation (MR), tricuspid regurgitation (TR), presentation with incessant VT, and preprocedural electrical storm (ES) were associated with increased risk of VT recurrence. Diabetes, implanted CRT, VT with hemodynamic instability, ES and advanced MR were the risk factors of mortality in women. ACEi/ARB use predicted a favorable outcome in both endpoints among females. In men, independent predictors of VT recurrence were the composite parameter of ES and multiple ICD therapies, presentation with incessant VT, severe MR, while independent predictors of mortality were age, LVEF, creatinine and previously implanted CRT.

**Conclusion:**

According to our investigation, there are pronounced sex differences in predictors of recurrence and mortality following VT ablation.

## Introduction

Ventricular tachycardia (VT) is one of the leading causes of sudden cardiac death (SCD) (1). Implantable cardioverter defibrillators (ICDs) effectively prevent SCD, while pharmacotherapy is routinely applied to reduce the number of arrhythmia episodes (2). However, antiarrhythmics are often ineffective, while ICD shocks increase mortality (3) and have a negative psychological impact (4). Therefore, radiofrequency catheter ablation (RFA) has become the gold standard therapeutic option. This modality has been proven superior to antiarrhythmic drug therapy (5) and might be the only solution in some electrical storm cases (6, 7).

Indications for catheter ablation in VT are clearly defined (2), and there is extensive literature available on factors that influence VT ablation success (8). However, female patients are underrepresented in the existing studies, therefore sex differences among predictors of VT recurrence or mortality following VT ablation are not known.

There are well-known differences between male and female patients in arrhythmology. As an example, atrioventricular nodal reentry tachycardia (AVNRT) is more common in females (9). Furthermore, VTs originating from the right ventricular outflow tract have different electro- anatomic characteristics (10), and male patients with arrhythmogenic right ventricular cardiomyopathy (ARVC) were shown to have larger areas with late potentials and higher rates of recurrence after catheter ablation (10). Ischemic heart disease, the major etiology of VT, shows marked sex differences regarding incidence and presentation. Specifically, ischemic heart disease is less common in women before their 50 s, generally has a worse prognosis, and more commonly presents in the form of microvascular dysfunction compared to overt large vessel disease (11). Moreover, non-ischemic VT etiology, which has worse overall prognosis (12), seems to be more prevalent in the female population (13).

Recent evidence suggests that women show a higher rate of 1-year recurrence after VT ablation, despite the more favorable baseline characteristics (14). Another study failed to confirm these findings and argued that sex itself does not influence the efficacy of catheter ablation (13). Therefore, while several predictors of VT recurrence have been described, there is an apparent lack of sex-specific data.

## Methods

### Patient population

Two-hundred ninety-nine patients were enrolled in our single-center, observational study, who underwent VT ablation between January 2005 and April 2021. Indication of catheter ablation was based on current ESC guidelines (2, 15) and EHRA expert consensus statement on catheter ablation of ventricular arrhythmias (16). The inclusion criteria were structural heart disease and detection of sustained, monomorphic VT with either a 12-lead ECG or an ICD. Structural heart disease was defined as a previous ischemic event or non-ischemic dilated cardiomyopathy (DCM). VT recurrence was defined as detection of a sustained, monomorphic VT episode with a 12-lead ECG, or any sustained ventricular tachycardia detected by an ICD. The exclusion criteria were idiopathic and polymorphic VTs. All patients agreed to the ablation procedure by giving a written, informed consent. Our study was approved by the Semmelweis University Ethics Committee and complied with the Declaration of Helsinki.

### Data collection

We collected the medical history, transthoracic echocardiographic parameters, and laboratory results prior to the first VT ablation. We also gathered the electrophysiological properties of the detected arrhythmia and VT ablation procedural data in a structured registry. We obtained the all-cause mortality status from the National Health Insurance Database of Hungary. The primary endpoints were 1-year all-cause mortality and VT recurrence within 1 year after catheter ablation.

### Echocardiography

We assessed the cardiac structure and function of each patient who underwent VT ablation before the procedure using transthoracic echocardiography (TTE). All the measurements were performed as recommended by the current guidelines of the American Society of Echocardiography (17, 18). In this study we measured the left ventricular end diastolic and end systolic diameters (LVEDD and LVESD) and right ventricular end diastolic diameter (RVEDD). Left ventricular ejection fraction (LVEF) was measured with the Simpson's method and right ventricular function was characterized by the tricuspid annular plane systolic excursion (TAPSE). We measured mitral or tricuspid valve regurgitation in a semiquantitative manner. Diastolic function was assessed using the transmitral flow velocities, E/A ratio and transmitral E wave deceleration time (DT) were calculated. We calculated the pulmonary artery systolic pressure (PASP) using the maximal velocity of the tricuspid regurgitation and estimated right atrial pressure, if applicable.

### Ablation procedure

We used right femoral venous access to insert a quadripolar diagnostic catheter (BIOTRONIK SE & Co. KG, Berlin, Germany) in the right ventricular apex, and a decapolar electrode (Bard, Boston Scientific Marlborough, Massachusetts, USA or St Jude Scientific, Saint Paul, Minnesota, USA) in the coronary sinus. For the insertion of the ablation catheter [Therapy Cool Path (St. Jude Scientific), Blazer Open-Irrigated (Boston Scientific), AlCath Black, AlCath Flux Blue (Biotronik), NaviStar ThermoCool, SmartTouch (Biosense Webster, Irvine, California, USA), TactiCath (Abbott, Chicago, Illinois, USA)] we used either a transvenous, a retrograde transaortic or a transseptal approach. For epicardial ablations the subxiphoid approach was used. In all cases, we used a 3D electroanatomical mapping system, which was connected to the ablation catheter (CARTO, Biosense Webster or EnSite, Abbott). To induce any significant VT, we delivered programmed extra-stimulation up to 3 beats. During hemodynamically tolerated VT we performed simultaneous activation and substrate mapping to identify the critical isthmus sites. In certain cases, we also used entrainment mapping to locate the critical sites of the reentrant circuit. Hemodynamically intolerable VTs were terminated with electrical cardioversion. Subsequently in these cases, substrate mapping was performed during sinus or ventricular paced rhythm. Myocardial scars were defined as a bipolar potential amplitude lower than 1.5 mV and dense scar areas were defined as lower than 0.5 mV with no local capture. The main ablation strategies were ablation of the critical isthmus and extensive substrate modification targeting local abnormal ventricular activities (LAVAs) and late potentials (LPs). Among hemodynamically stable patients, only substrate modification was performed. The only isthmus-imaging modalities used were activation and substrate mapping. The primary aim of the procedure was to eliminate all inducible clinically significant VTs. Acute procedural success was defined as VT non-inducibility after the procedure with programmed extra stimulation up to 3 beats. If the VT was not inducible during the procedure, the procedural success was defined as complete elimination of all LAVAs and LPs.

### Statistical analysis

We used SPSS version 27 (Apache Software Foundation, Forest Hill, MD, USA) for the statistical analysis. The categorical variables are listed as event numbers and percentages and the continuous variables are presented as median and 25 and 75% interquartile ranges. We used Kolmogorov-Smirnov's test to identify variables that follow a normal distribution. To compare the two patient populations, we performed Chi-squared test for dichotomous variables and Mann-Whitney test for continuous variables. For ordinal variables, we used the Kruskal- Wallis *H* test. To identify the variables that correlate with our primary endpoints, we used univariate Cox regression analysis. Scalar variables were converted to Z-scores. We also assessed the correlation between the individual variables. Multicollinearity was determined by variance inflation factor (VIF) values together with normal probability plots. The variables we found significant in the univariate Cox model and < 5 VIF were included in the multivariate analysis to identify the independent predictors of the primary endpoints. The results are reported in hazard ratios with 95% confidence intervals. To visualize our findings, we conducted a Kaplan-Meier survival analysis using the Python software (Python Software Foundation). To include scalar values in our Kaplan-Meier analysis, we performed receiver operating characteristics curve (ROC) analysis and identified the optimal cut-off point of the certain variable with Youden's index. Results with a *p*-value < 0.05 were considered statistically significant.

## Results

Out of the 299 patients, 34 (11.4%) were female. Ninety-three male (35.36%) and 13 female (38.24%) patients experienced VT recurrence within 1 year after ablation., while 50 men (19.01%) and 11 women (32.35%) died during the follow-up. No significant difference was found between women and men in terms of 1-year VT recurrence (*p* = 0.74) or 1-year all-cause mortality (*p* = 0.07). The median age was 68 years in the male and 69 years in the female population. The most common comorbidities were hypertension (74%), chronic heart failure (82%) and coronary artery disease (82%). Beta-blockers (90%) and ACEis/ARBs (83%) were the most frequently prescribed medications. We treated 203 patients (68%) with amiodarone before ablation. After the procedure 205 (69%) patients remained on amiodarone treatment. Women more frequently had severe tricuspid regurgitation (TR) (*p* = 0.02), while men had higher hemoglobin (*p* = 0.03) and creatinine levels (*p* = 0.014), however, the respective median values of these parameters were in normal ranges (Table 1).

**Table 1:** Baseline parameters of the study population.

**Baseline parameters**	**Male**	**Female**	***P*-value**
Age (year)	68 [58.3–74.6]	69 [63.5–72.6]	0.29
BMI	27.78 [25.1–31.6]	27.35 [26.5–32.2]	0.95
Atrial fibrillation	81 (30.8%)	10 (29.41%)	0.87
Hypertension	193 (73.38%)	28 (82.35%)	0.26
Diabetes	84 (31.94%)	6 (17.65%)	0.09
COPD	30 (11.41%)	6 (17.65%)	0.29
SCD	38 (14.5%)	5 (14.71%)	0.97
CAD	218 (82.89%)	25 (73.53%)	0.18
PCI	109 (41.44%)	10 (30.3%)	0.22
CABG	59 (22.43%)	3 (9.09%)	0.11
AMI	192 (73.0%)	22 (64.71%)	0.31
ICD	203 (77.19%)	24 (70.59%)	0.39
CRT	65 (24.71%)	7 (20.59%)	0.60
NYHA ≥3	84 (31.94%)	10 (29.41%)	0.77
DCM	187 (71.37%)	20 (60.61%)	0.20
iDCM	155 (82%)	13 (65%)	0.07
niDCM	34 (18%)	7 (35%)	0.07
LVEF (%)	34 [25.0–42.0]	32 [27.0–42.0]	0.96
LVEDD (mm)	60 [55.0–68.0]	59 [52.0–64.0]	0.17
LVESD (mm)	49 [42.0–57.0]	49.5 [40.8–56.2]	0.58
RVEDD (mm)	36 [32.0–40.0]	34 [30.8–39.2]	0.19
TAPSE (mm)	19 [15.8–23.0]	17 [14.5–20.0]	0.14
E/A ratio	1.01 [0.7–1.8]	1.08 [0.8–1.9]	0.42
DT (ms)	165 [140.0–219.0]	152 [127.5–194.8]	0.15
PASP (mmHg)	37 [29.0–45.8]	38 [32.0–49.0]	0.29
MR 1–2	167 (69.58%)	15 (53.57%)	0.09
MR 3–4	54 (22.5%)	10 (35.71%)	0.12
TR 1–2	177 (75.0%)	18 (64.29%)	0.22
TR 3–4	23 (9.75%)	7 (25.0%)	**0.02**
LV aneurysm	59 (24.48%)	8 (26.67%)	0.79
Hemoglobin (g/L)	137 [123.0–148.0]	122 [113.5–141.5]	**0.03**
Creatinine (μmol/L)	105 [88.0–139.0]	88 [76.5–119.0]	**0.014**
GFR (ml/min/1.73m^2^)	62 [45.4–82.4]	57 [40.3–76.8]	0.53
Urea (mmol/L)	7.85 [6.0–11.0]	7.6 [6.4–12.6]	0.95
Glucose (mmol/L)	6.2 [5.3–7.4]	6.6 [5.9–8.8]	0.17
Furosemide	156 (59.32%)	21 (63.64%)	0.63
ACEi/ARB	220 (86.96%)	25 (83.33%)	0.58
Statin	193 (73.38%)	22 (66.67%)	0.41
MRA	143 (54.58%)	18 (54.55%)	0.99
Amiodarone	182 (68.67%)	21 (63.64%)	0.52
Beta blocker	238 (90.49%)	29 (90.62%)	0.99
AAD resistance	178 (67.68%)	24 (72.73%)	0.56
Amiodarone at discharge	183 (69.05%)	22 (64.70%)	0.45
ICD shock	118 (45.04%)	11 (33.33%)	0.20
HD instability	102 (38.78%)	14 (41.18%)	0.79
Incessant VT	94 (35.74%)	11 (32.35%)	0.70
Electrical storm	33 (12.6%)	7 (20.59%)	0.20
Cumulative ICD therapies	108 (41.22%)	14 (41.18%)	0.99
Electrical storm/incessant VT	103 (39.16%)	14 (41.18%)	0.82
Electrical storm/cumulative ICD therapies	126 (47.54%)	16 (47.05%)	0.96
Transseptal	41 (15.77%)	4 (12.12%)	0.80
Transaortic	218 (83.85%)	26 (78.79%)	0.46
Epicardial	31 (11.83%)	4 (11.76%)	0.99
Procedure time (min)	90 [70.0–120.0]	92 [81.2–108.8]	0.73
Radiation duration (min)	9 [5.8–14.5]	8 [6.0–15.4]	0.95
Inducible clinical VT	206 (78.33%)	27 (84.38%)	0.43
≥1 inducible morphology	229 (87.07%)	29 (90.62%)	0.78
VTCL (ms)	398 [340.0–450.0]	385 [350.0–482.5]	0.64
Elimination of all inducible VTs	202 (79.53%)	29 (87.88%)	0.35
Complications	33 (12.6%)	7 (20.59%)	0.20
Recurrence (1-year)	93 (35.36%)	13 (38.24%)	0.74
Reablation	45 (17.11%)	6 (17.65%)	0.94
All-cause mortality (1-year)	50 (19.01%)	11 (32.35%)	0.07

Values are presented as median [25th−75th interquartile range] or as event numbers (%). *P* < 0.05 considered significant. BMI, body mass index; COPD, chronic obstructive pulmonary disease; SCD, sudden cardiac death; CAD, coronary artery disease; PCI, percutaneous coronary intervention; CABG, coronary artery bypass grafting; AMI, acute myocardial infarction; ICD, implantable cardioverter defibrillator; CRT, implanted cardiac resynchronization therapy device; DCM, dilated cardiomyopathy; iDCM, ischemic dilated cardiomyopathy; niDCM, non-ischemic dilated cardiomyopathy; NYHA, New York Heart Association functional class prior to the ablation; LVEF, left ventricular ejection fraction; LVEDD, left ventricular end diastolic diameter; LVESD, left ventricular end systolic diameter; RVEDD, right ventricular end diastolic diameter; TAPSE, tricuspid annular plane systolic excursion; E/A, transmitral E and A wave ratio; DT, transmitral E wave deceleration time; MR, mitral regurgitation; TR, tricuspid regurgitation; LV, left ventricle; PASP, pulmonary artery systolic pressure; GFR, glomerulus filtration rate; MRA, mineralocorticoid-receptor antagonist therapy; ACEi/ARB, angiotensin-convertase enzyme inhibitor or angiotensin-receptor blocker therapy, AAD, anti-arrhythmic drug; HD, hemodynamic; VT, ventricular tachycardia; VTCL, ventricular tachycardia cycle length.

We used univariate Cox regression analysis to assess the predictors of 1-year recurrence and 1-year all-cause mortality in both sexes (Tables 2, 3).

**Table 2:** Sex-specific predictors of ventricular tachycardia recurrence with univariate Cox regression analysis.

**Recurrence univariates**	**Men**		**Women**	
	**HR (95% CI)**	***P*-value**	**HR (95% CI)**	***P*-value**
Age (year)	1.04 (0.85–1.28)	0.69	0.89 (0.46–1.75)	0.74
BMI	0.92 (0.71–1.19)	0.52	1.38 (0.66–2.89)	0.39
Atrial fibrillation	1.04 (0.85–1.27)	0.70	1.6 (0.97–2.64)	0.07
Hypertension	0.86 (0.71–1.05)	0.13	0.67 (0.42–1.05)	0.08
Diabetes	1.06 (0.87–1.3)	0.55	1.06 (0.6–1.88)	0.85
COPD	1.03 (0.84–1.26)	0.77	0.66 (0.3–1.44)	0.30
SCD	0.99 (0.8–1.22)	0.90	1.11 (0.65–1.9)	0.70
CAD	0.97 (0.79–1.18)	0.75	0.74 (0.45–1.22)	0.24
PCI	0.92 (0.75–1.13)	0.41	0.67 (0.33–1.33)	0.25
CABG	1.1 (0.9–1.33)	0.36	0.9 (0.5–1.61)	0.72
AMI	0.94 (0.77–1.15)	0.55	0.76 (0.45–1.29)	0.31
ICD	1.22 (0.97–1.54)	0.09	1.12 (0.62–2.01)	0.71
CRT	1.29 (1.07–1.55)	**0.007**	1.23 (0.73–2.08)	0.44
NYHA ≥3	1.24 (1.02–1.5)	**0.03**	1.38 (0.82–2.3)	0.22
DCM	1.44 (1.13–1.84)	**0.003**	1.01 (0.59–1.75)	0.96
iDCM	0.76 (0.44–1.31)	0.32	0.41 (0.10–1.64)	0.21
niDCM	1.33 (0.77–2.31)	0.31	2.46 (0.61–9.88)	0.21
LVEF (%)	0.76 (0.61–0.94)	**0.01**	0.73 (0.4–1.33)	0.30
LVEDD (mm)	1.29 (1.06–1.57)	**0.01**	1.19 (0.62–2.26)	0.60
LVESD (mm)	1.35 (1.11–1.64)	**0.003**	1.69 (0.78–3.65)	0.18
RVEDD (mm)	1.04 (0.85–1.28)	0.69	1.67 (0.9–3.11)	0.10
TAPSE (mm)	0.87 (0.69–1.1)	0.25	0.73 (0.38–1.41)	0.35
E/A ratio	1.06 (0.83–1.36)	0.63	1.28 (0.73–2.26)	0.39
DT (ms)	0.91 (0.72–1.14)	0.41	0.51 (0.18–1.43)	0.20
PASP (mmHg)	1.08 (0.86–1.35)	0.53	1.12 (0.64–1.95)	0.69
MR 1–2	0.88 (0.72–1.08)	0.22	0.47 (0.24–0.93)	**0.03**
MR 3–4	1.35 (1.13–1.62)	**0.001**	2.06 (1.12–3.81)	**0.02**
TR 1–2	1.15 (0.91–1.43)	0.24	0.42 (0.22–0.81)	**0.01**
TR 3–4	0.98 (0.79–1.22)	0.85	1.91 (1.11–3.29)	**0.02**
LV aneurysm	0.84 (0.67–1.06)	0.14	1.06 (0.59–1.91)	0.84
Hemoglobin (g/L)	0.82 (0.66–1.02)	0.07	0.81 (0.45–1.44)	0.47
Creatinine (μmol/L)	0.96 (0.77–1.21)	0.76	0.96 (0.54–1.74)	0.90
GFR (ml/min/1.73m^2^)	1.04 (0.84–1.3)	0.70	1.14 (0.67–1.94)	0.64
Glucose (mmol/L)	1.01 (0.82–1.25)	0.90	0.85 (0.41–1.77)	0.67
Furosemide	1.46 (1.17–1.82)	**0.0009**	1.15 (0.64–2.05)	0.64
ACEi/ARB	0.9 (0.74–1.09)	0.29	0.49 (0.3–0.79)	**0.004**
Statin	1.07 (0.86–1.32)	0.54	0.65 (0.38–1.11)	0.11
MRA	1.18 (0.96–1.45)	0.11	1.8 (0.94–3.46)	0.08
Amiodarone	1.22 (0.98–1.52)	0.08	0.76 (0.44–1.31)	0.32
Beta blocker	0.91 (0.75–1.11)	0.35	105.52 (0.0–inf)	0.99
AAD resistance	1.17 (0.94–1.44)	0.16	1.08 (0.61–1.94)	0.79
ICD shock	1.36 (1.1–1.67)	**0.004**	1.37 (0.79–2.35)	0.26
HD instability	1.21 (0.99–1.47)	0.07	1.57 (0.92–2.71)	0.10
Incessant VT	1.26 (1.04–1.54)	**0.02**	1.43 (0.86–2.38)	0.18
Electrical storm	1.12 (0.93–1.36)	0.22	1.53 (0.94–2.48)	0.08
Cumulative ICD therapies	1.33 (1.09–1.63)	**0.005**	1.36 (0.8–2.33)	0.26
Electrical storm/incessant VT	1.26 (1.04–1.54)	**0.02**	1.83 (1.05–3.19)	**0.03**
Electrical storm/cumulative ICD therapies	2.05 (1.35–3.10)	**0.001**	1.48 (0.50–4.39)	0.49
Transseptal	0.91 (0.73–1.14)	0.43	0.0 (0.0–inf)	0.99
Transaortic	1.02 (0.83–1.26)	0.85	1.27 (0.68–2.36)	0.46
Epicardial	1.11 (0.92–1.34)	0.28	0.88 (0.45–1.69)	0.69
Procedure time (min)	1.27 (0.98–1.64)	0.07	1.27 (0.56–2.9)	0.57
Radiation duration (min)	1.16 (0.89–1.51)	0.29	1.54 (0.61–3.93)	0.36
Inducible clinical VT	1.47 (1.12–1.92)	**0.006**	1.34 (0.64–2.81)	0.44
≥1 inducible morphology	2.03 (1.27–3.24)	**0.003**	1.02 (0.56–1.85)	0.95
VTCL (ms)	1.17 (0.96–1.44)	0.13	1.17 (0.64–2.16)	0.61
Elimination of all inducible VTs	1.03 (0.84–1.28)	0.76	0.66 (0.43–1.01)	0.06

*P* < 0.05 considered significant. BMI, body mass index; COPD, chronic obstructive pulmonary disease; SCD, sudden cardiac death; CAD, coronary artery disease; PCI, percutaneous coronary intervention; CABG, coronary artery bypass grafting; AMI, acute myocardial infarction; ICD, implantable cardioverter defibrillator; CRT, implanted cardiac resynchronization therapy device; DCM, dilated cardiomyopathy; iDCM, ischemic dilated cardiomyopathy; niDCM, non-ischemic dilated cardiomyopathy; NYHA, New York Heart Association functional class prior to the ablation; LVEF, left ventricular ejection fraction; LVEDD, left ventricular end diastolic diameter; LVESD, left ventricular end systolic diameter; RVEDD, right ventricular end diastolic diameter; TAPSE, tricuspid annular plane systolic excursion; E/A, transmitral E and A wave ratio; DT, transmitral E wave deceleration time; MR, mitral regurgitation; TR, tricuspid regurgitation; LV, left ventricle; PASP, pulmonary artery systolic pressure; GFR, glomerulus filtration rate; MRA, mineralocorticoid-receptor antagonist therapy; ACEi/ARB, angiotensin-convertase enzyme inhibitor or angiotensin-receptor blocker therapy, AAD, anti-arrhythmic drug; HD, hemodynamic; VT, ventricular tachycardia; VTCL, ventricular tachycardia cycle length. Bold values indicate statistically significant results.

**Table 3:** Sex-specific predictors of all-cause mortality with univariate Cox regression analysis.

**Mortality univariates**	**Men**		**Women**	
	**HR (95% CI)**	***P*-value**	**HR (95% CI)**	***P*-value**
Age (year)	1.66 (1.19–2.33)	**0.003**	1.2 (0.59–2.44)	0.62
BMI	0.96 (0.68–1.36)	0.84	1.4 (0.74–2.65)	0.30
Atrial fibrillation	1.18 (0.9–1.53)	0.22	1.21 (0.69–2.11)	0.51
Hypertension	0.91 (0.7–1.19)	0.49	0.7 (0.42–1.17)	0.17
Diabetes	1.09 (0.83–1.42)	0.54	1.77 (1.1–2.84)	**0.02**
COPD	1.23 (0.98–1.55)	0.07	0.75 (0.34–1.63)	0.46
SCD	1.13 (0.87–1.45)	0.36	1.16 (0.67–2.0)	0.59
CAD	1.18 (0.86–1.63)	0.31	0.98 (0.55–1.76)	0.94
PCI	1.07 (0.81–1.41)	0.62	1.23 (0.7–2.16)	0.48
CABG	1.19 (0.92–1.53)	0.19	0.01 (0.0–inf)	0.99
AMI	1.14 (0.84–1.53)	0.40	0.99 (0.55–1.79)	0.98
ICD	1.14 (0.84–1.54)	0.41	0.7 (0.41–1.2)	0.20
CRT	1.47 (1.15–1.87)	**0.002**	1.86 (1.15–3.01)	**0.01**
NYHA ≥3	1.27 (0.98–1.65)	0.07	1.01 (0.55–1.85)	0.98
DCM	1.35 (0.97–1.86)	0.07	1.08 (0.59–1.96)	0.81
iDCM	1.31 (0.55–3.12)	0.54	0.67 (0.15–2.99)	0.60
niDCM	0.77 (0.32–1.83)	0.55	1.50 (0.34–6.72)	0.60
LVEF (%)	0.58 (0.42–0.8)	**0.001**	1.13 (0.64–2.0)	0.67
LVEDD (mm)	1.31 (1.0–1.71)	0.054	1.39 (0.7–2.74)	0.35
LVESD (mm)	1.41 (1.06–1.87)	**0.02**	1.26 (0.62–2.59)	0.52
RVEDD (mm)	1.08 (0.83–1.42)	0.56	0.87 (0.46–1.65)	0.67
TAPSE (mm)	0.74 (0.54–1.0)	0.053	0.87 (0.45–1.68)	0.67
E/A ratio	1.35 (0.99–1.84)	0.06	0.96 (0.5–1.85)	0.90
DT (ms)	0.46 (0.3–0.68)	**0.0001**	0.64 (0.25–1.61)	0.34
PASP (mmHg)	1.16 (0.85–1.59)	0.35	0.92 (0.5–1.69)	0.78
MR 1–2	1.05 (0.79–1.4)	0.74	0.45 (0.23–0.88)	**0.02**
MR 3–4	1.16 (0.89–1.5)	0.26	2.2 (1.21–3.99)	**0.01**
TR 1–2	1.15 (0.85–1.55)	0.38	0.92 (0.51–1.66)	0.79
TR 3–4	1.07 (0.83–1.38)	0.59	1.12 (0.63–1.99)	0.70
LV aneurysm	1.05 (0.8–1.38)	0.74	1.27 (0.74–2.18)	0.39
Hemoglobin (g/L)	0.8 (0.63–1.02)	0.07	0.69 (0.37–1.3)	0.26
Creatinine (μmol/L)	1.27 (1.09–1.49)	**0.003**	1.0 (0.55–1.83)	0.99
GFR (ml/min/1.73 m^2^)	0.64 (0.46–0.89)	**0.007**	1.03 (0.58–1.81)	0.93
Glucose (mmol/L)	1.06 (0.82–1.38)	0.65	2.94 (1.47–5.86)	**0.002**
Furosemide	1.72 (1.23–2.42)	**0.002**	1.23 (0.64–2.36)	0.53
ACEi/ARB	1.2 (0.85–1.69)	0.31	0.5 (0.31–0.81)	**0.005**
Statin	1.06 (0.8–1.42)	0.68	0.82 (0.45–1.49)	0.52
MRA	1.58 (1.16–2.17)	**0.004**	1.2 (0.64–2.26)	0.56
Amiodarone	1.07 (0.81–1.42)	0.64	1.19 (0.62–2.27)	0.61
Beta blocker	0.98 (0.75–1.29)	0.90	0.96 (0.53–1.77)	0.90
AAD resistance	1.16 (0.86–1.56)	0.32	1.21 (0.61–2.41)	0.59
ICD shock	1.16 (0.88–1.53)	0.29	1.56 (0.87–2.79)	0.14
HD instability	1.0 (0.76–1.32)	0.99	2.3 (1.19–4.43)	**0.01**
Incessant VT	1.27 (0.97–1.66)	0.08	1.33 (0.76–2.31)	0.32
Electrical storm	1.21 (0.95–1.53)	0.13	1.87 (1.15–3.02)	**0.01**
Cumulative ICD therapies	1.21 (0.92–1.6)	0.16	0.92 (0.5–1.69)	0.79
Electrical storm/incessant VT	1.29 (0.98–1.69)	0.07	1.76 (0.96–3.22)	0.07
Electrical storm/cumulative ICD therapies	2.01 (1.13–3.55)	**0.02**	1.49 (0.46–4.90)	0.51
Transseptal	1.2 (0.95–1.52)	0.13	0.95 (0.48–1.86)	0.88
Transaortic	0.84 (0.66–1.07)	0.15	1155.61 (0.0–inf)	0.99
Epicardial	1.03 (0.78–1.35)	0.86	1.25 (0.76–2.05)	0.38
Procedure time (min)	1.18 (0.84–1.66)	0.35	0.94 (0.36–2.45)	0.91
Radiation duration (min)	1.23 (0.87–1.74)	0.23	1.38 (0.53–3.61)	0.51
Inducible clinical VT	1.04 (0.78–1.38)	0.78	1.15 (0.54–2.44)	0.72
≥1 inducible morphology	1.11 (0.81–1.51)	0.53	0.91 (0.5–1.67)	0.76
VTCL (ms)	1.3 (0.97–1.74)	0.08	0.67 (0.3–1.52)	0.34
Elimination of all inducible VTs	1.04 (0.78–1.4)	0.78	1.07 (0.54–2.09)	0.85
Complications	1.14 (0.89–1.46)	0.31	0.94 (0.51–1.75)	0.84

*P* < 0.05 considered significant. BMI, body mass index; COPD, chronic obstructive pulmonary disease; SCD, sudden cardiac death; CAD, coronary artery disease; PCI, percutaneous coronary intervention; CABG, coronary artery bypass grafting; AMI, acute myocardial infarction; ICD, implantable cardioverter defibrillator; CRT, implanted cardiac resynchronization therapy device; DCM, dilated cardiomyopathy; iDCM, ischemic dilated cardiomyopathy; niDCM, non-ischemic dilated cardiomyopathy; NYHA, New York Heart Association functional class prior to the ablation; LVEF, left ventricular ejection fraction; LVEDD, left ventricular end diastolic diameter; LVESD, left ventricular end systolic diameter; RVEDD, right ventricular end diastolic diameter; TAPSE, tricuspid annular plane systolic excursion; E/A, transmitral E and A wave ratio; DT, transmitral E wave deceleration time; MR, mitral regurgitation; TR, tricuspid regurgitation; PASP, pulmonary artery systolic pressure; GFR, glomerulus filtration rate; MRA, mineralocorticoid-receptor antagonist therapy; ACEi/ARB, angiotensin-convertase enzyme inhibitor or angiotensin-receptor blocker therapy, AAD, anti-arrhythmic drug; HD, hemodynamic; VT, ventricular tachycardia; VTCL, ventricular tachycardia cycle length. Bold values indicate statistically significant results.

Among women, a composite parameter of presentation with incessant VT and electrical storm (ES) was associated with increased risk of VT recurrence [1.83 (1.05–3.19), *p* = 0.03], while angiotensin convertase enzyme inhibitor/angiotensin receptor blocker (ACEi/ARB) use predicted lower recurrence rates [0.49 (0.30–0.79), *p* = 0.004]. Advanced DCM characterized by more severe tricuspid regurgitation (TR) or mitral regurgitation (MR) showed increased risk of VT recurrence [1.91 (1.11–3.29), *p* = 0.02 and 2.06 (1.12–3.81), *p* = 0.02, respectively] and in case of MR, higher risk of death as well [2.20 (1.21–3.99), *p* = 0.01]. ACEi/ARB therapy [0.50 (0.31–0.81), *p* = 0.005] was also associated with better survival in the female population. Meanwhile, an implanted cardiac resynchronization therapy (CRT) device [1.86 (1.15–3.01), *p* = 0.02], presence of diabetes [1.77 (1.10–2.84), *p* = 0.02] and VT causing hemodynamic instability [2.30 (1.19–4.43), *p* = 0.01] or ES [1.87 (1.15–3.02), *p* = 0.01] were identified as risk factors of mortality.

**Figure 1:**
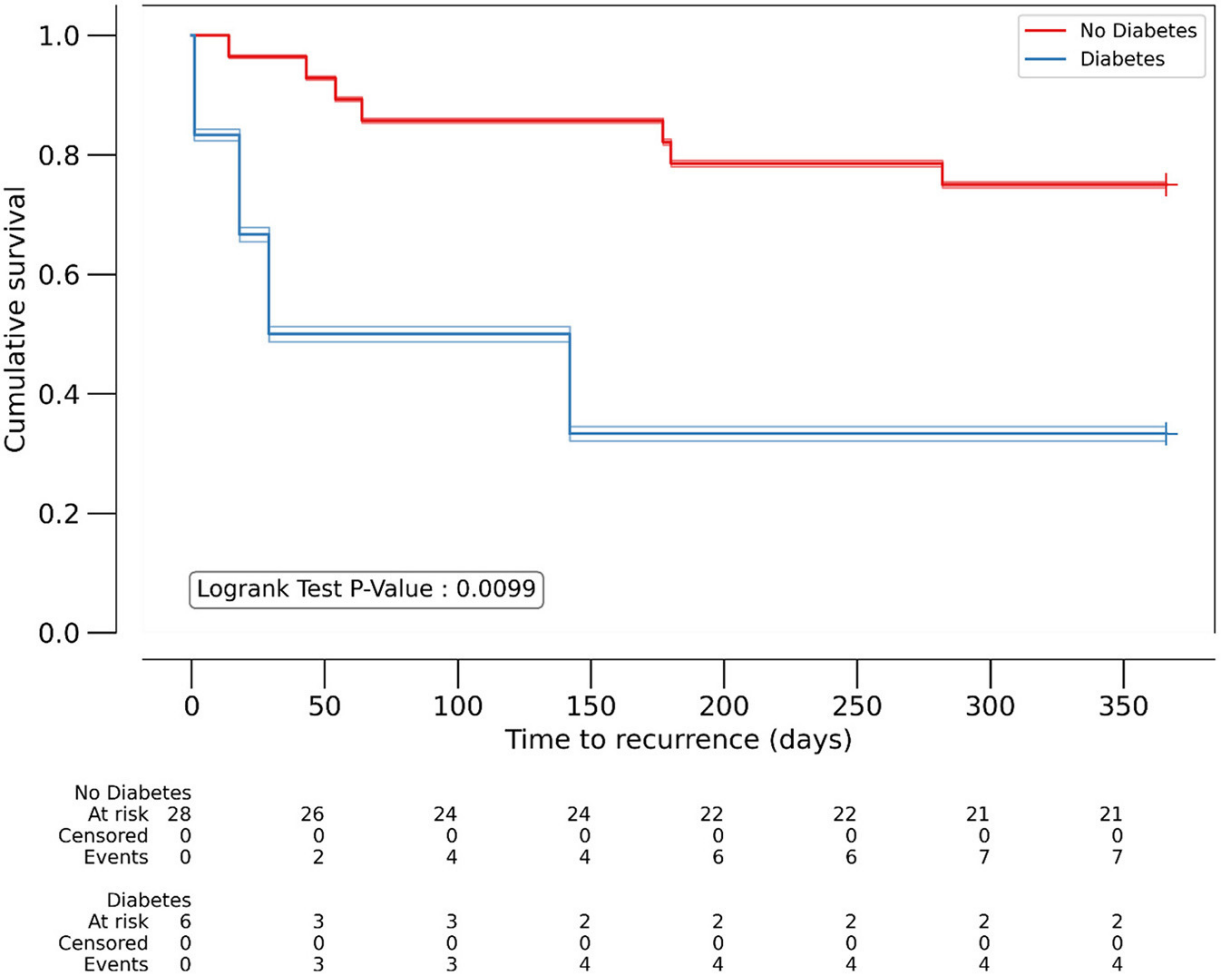
Kaplan-Meyer analysis of diabetes as predictor of 1-year all-cause mortality in women. *P* < 0.05 considered significant. Number of patients at risk at each 50-day timepoint are reported above x-axis for the individual patient groups.

**Figure 2:**
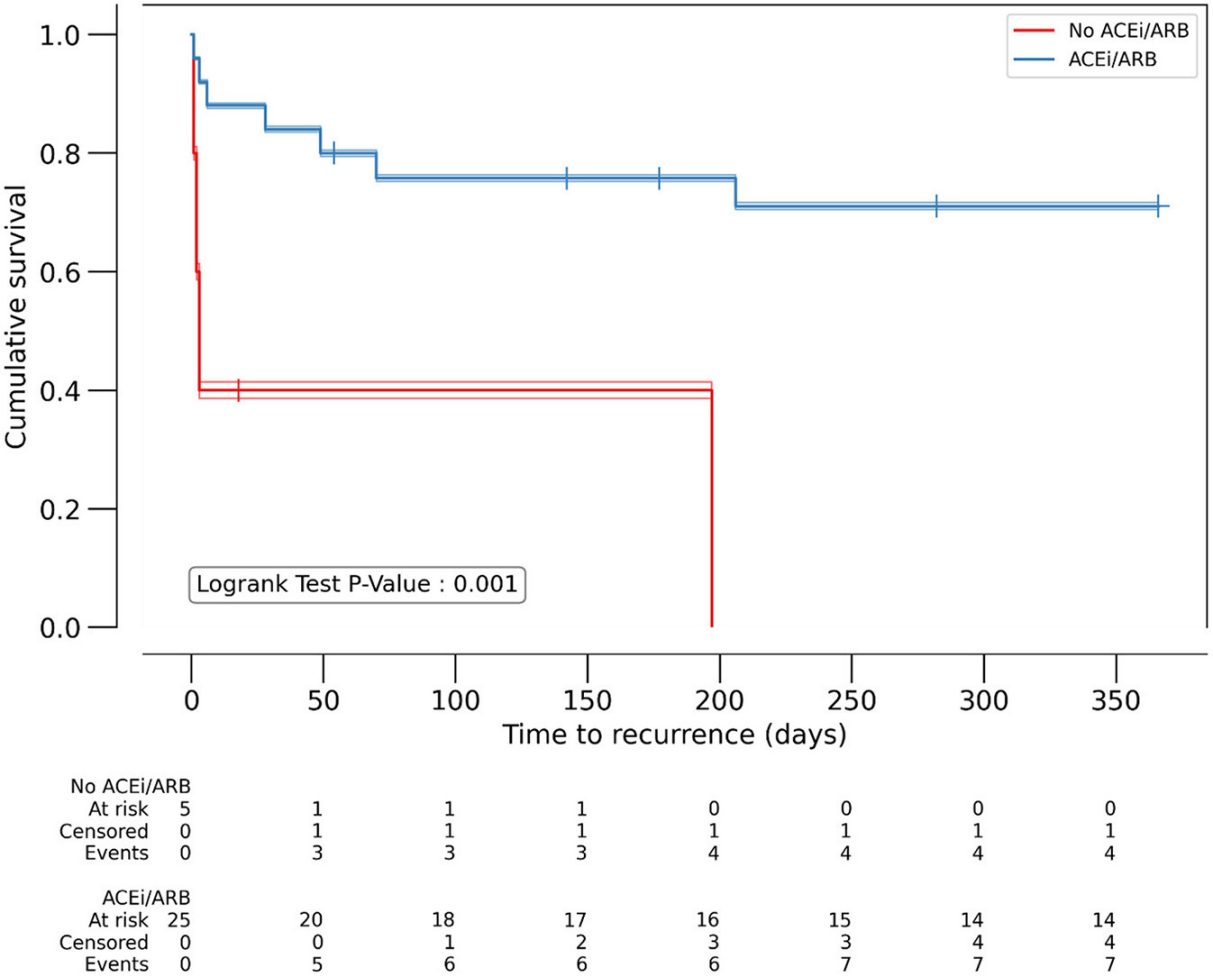
Kaplan-Meyer analysis of ACEi/ARB as predictor of 1-year VT recurrence in women. *P* < 0.05 considered significant. ACEi/ARB: angiotensin convertase enzyme inhibitor or angiotensin receptor blocker therapy. Number of patients at risk at each 50- day timepoint are reported above x-axis for the individual patient groups.

Predictors of mortality among women were visualized using Kaplan-Meier survival analysis, which shows significantly worse outcome in patients with diabetes (*p* = 0.01), while ACEi/ARB intake was associated with lower recurrence rate (*p* = 0.001) (Figures 1, 2).

In males, univariate analysis regarding 1-year VT recurrence identified several predictors (Table 2). Indicators of malignant arrhythmia behavior, such as a composite parameter of preprocedural ES and multiple ICD therapies [2.18 (1.41–3.37), *p* = 0.0004], and presentation with incessant VT [1.55 (1.01–2.37), *p* = 0.044] predicted 1-year VT recurrence independently. Furthermore, severely enlarged mitral annulus leading to more severe MR [2.09 (1.32–3.32), *p* = 0.002] was also independently associated with higher risk of recurrence (Table 4).

**Table 4:** Independent predictors of ventricular tachycardia recurrence in males.

**Recurrence multivariate parameters**	**HR (95% CI)**	***P*-value**
LVEF (%)	0.86 (0.69–1.09)	0.21
MR 3–4	2.09 (1.32–3.32)	**0.002**
Electrical storm/cumulative ICD therapies	2.18 (1.41–3.37)	**0.0004**
Incessant VT	1.55 (1.01–2.37)	**0.044**

*P* < 0.05 considered significant. LVEF, left ventricular ejection fraction; MR, mitral regurgitation; ICD, implantable cardioverter defibrillator; VT, ventricular tachycardia. Bold values indicate statistically significant results.

Regarding 1 year mortality, we found that those who were older [1.45 (1.03–2.04), *p* = 0.03], had lower LVEF [0.65 (0.44–0.96), *p* = 0.029], had impaired kidney function indicated by higher creatinine levels [1.26 (1.03–1.53), *p* = 0.025] and had a previously implanted CRT device [1.85 (1.01–3.83), *p* = 0.048] were at higher risk of death within 1 year (Table 5).

**Table 5:** Independent predictors of all-cause mortality in males.

**Mortality multivariate parameters**	**HR (95% CI)**	***P*-value**
Age (year)	1.45 (1.03–2.04)	0.03
CRT	1.85 (1.01–3.83)	0.048
LVEF (%)	0.65 (0.44–0.96)	0.029
Creatinine (μmol/L)	1.26 (1.03–1.53)	0.025

*P* < 0.05 considered significant. CRT, cardiac resynchronization therapy; LVEF, left ventricular ejection fraction.

We observed 42 complication episodes in 40 patients. Eighteen patients had vascular complications related to the punctures, 14 of them required vascular surgery. In 17 patients we saw accumulated pericardial fluid, in 12 of 17 cases a pericardial puncture and drainage was necessary. Four patients had transient ischemic attack/stroke after ablation, while the 3 remaining complications were an acute heart failure episode, third degree AV block and electromechanical dissociation requiring brief resuscitation and intraaortic balloon pump implantation.

## Discussion

In our study population, we could identify significant sex differences among the predictive factors for 1-year VT recurrence and 1-year mortality. Since female patients are often underrepresented in previous clinical studies, such data is of special importance. In our analysis, we found no differences in 1-year all-cause mortality or 1-year VT recurrence between men and women. We identified severe mitral regurgitation as general VT recurrence predictor, while a previously implanted CRT device predicted higher mortality rate in both sexes. ES—either as a single or a composite parameter—correlated with worse outcomes in both male and female patients in both endpoints.

History of diabetes, an implanted CRT device, advanced MR, prior ES and HD instability during VT were sex- specific predictors of 1-year mortality in women. ACEi/ARB therapy was associated with better outcomes, while advanced MR predicted worse outcomes regarding both endpoints. Due to the insufficient number of events among females, we could not perform a multivariate analysis to identify the independent predictors.

There is existing literature about sex-related differences in susceptibility to ventricular arrhythmias (19). The most studied mechanisms involve differences in ion channel density or gene- and protein expression of certain ion channels causing alterations in intracellular calcium handling and consequently in ventricular repolarization. However, existing evidence on VT-related sex-differences in structurally abnormal hearts is scarce.

Russo et al. analyzed the data from the MUSTT trial examining patients with coronary artery disease, and found that women were older, more likely had severe, recently diagnosed coronary artery disease and heart failure compared to men, while less commonly had inducible sustained VT during electrophysiological study. Despite the prevalent risk factors among females, they had less inducible VTs, which suggests that male sex itself might be a VT risk factor. However, due to the heavy male-majority in the study, these findings can be interpreted only as a hypothesis-generating (20).

In the subgroup analysis of the MADIT-II trial men with previous myocardial infarction had more benefit from primary prevention ICD compared to conventional therapy, however this difference was not present in women (21). According to the results of the Danish ICD registry, male sex was a risk factor of both appropriate and inappropriate therapy in patients with ischemic heart disease (22). Similarly, in a MADIT-CRT sub-study, women suffered less VT or ventricular fibrillation (VF) episodes or sudden cardiac death. These findings were more prominent among women with ischemic cardiomyopathy. It is important to note, that appropriate shock was a strong predictor of death in women (23, 24).

Several other trials also suggest that women are less susceptible to ventricular arrhythmias compared to men in this specific patient population. Generally, women are underrepresented in these studies, which is certainly a limitation, however, as a recurring result from multiple, independent studies it is reasonable to hypothesize that sex differences are present among these patients.

It is well-known that women comprise a higher proportion of patients in the non-ischemic group. However, even within this subgroup, the low absolute case numbers still limit the interpretation of the observations (25).

The existing literature is controversial about sex differences among patients undergoing VT ablation. Frankel and colleagues found higher VT recurrence rates after ablation among women (14), while other studies failed to confirm sex as an independent predictor (13). This latter study found that age, LVEF, NYHA class and VT recurrence, but not the sex were associated with increased mortality risk. This is in line with our finding that sex itself did not determine the outcome of the catheter ablation procedure. Importantly, the above-mentioned studies focused on sex differences in outcomes and did not aim to identify sex-specific predictors of success and outcome.

In our analysis we identified diabetes as sex-specific risk factors of postprocedural 1-year mortality in women. Diabetes is a well-known risk factor of heart failure and mortality in women with CAD (11) which is the dominant VT etiology in our population. It appears that similarly to ischemic heart disease, diabetes is a risk factor of mortality also in females undergoing VT ablation.

Another sex-specific finding in our investigation, the ACEi/ARB therapy, correlated with a lower risk of both endpoints in women. Since our study population mostly consists of patients with ischemic heart disease, the consequent heart failure predominantly contributes to mortality. ACE inhibitors are known to prevent, decrease and in some cases reverse pathological myocardial remodeling. These effects might also promote mechanisms inside the scar leading to lower recurrence rates.

These results show that among diabetic females, who do not tolerate ACEi/ARB, an invasive approach should be carefully deliberated and close post procedural follow-up may be necessary.

Predictors of recurrence in men are based on two concepts. Firstly, in dilated cardiomyopathy patients, MR severity indicates advanced cardiac remodeling. Secondly, a group of factors indicating a more arrhythmogenic VT substrate includes presentation with incessant VT, cumulative ICD therapies and ES. Our findings show that severe DCM and substrate complexity reduce long-term procedural success.

Meanwhile, independent predictors of 1-year mortality were older age, lower LVEF, implanted CRT and higher creatinine level. Since heart failure is a common comorbidity in our patient group, LVEF as an independent predictor emphasizes the importance of appropriate, individualized heart failure management. The predictive value of a previously implanted CRT and higher creatinine level is in line with this finding, and it indicates that VT ablation in patients with advanced heart failure carries a substantially higher risk of death.

Our findings in the male population are primarily in line with previous non-sex-specific literature (26); this also supports the assumption that existing data can be correctly applied to male patients but not necessarily to females.

It is also important to note, that complication rate did not differ between men and women.

Our study population represents real-world data derived from our registry, however, as widely known, women are underrepresented in this population, therefore our results should be interpreted cautiously. Doubtlessly, only a well-designed, prospective trial could provide firm evidence about the appropriate management of these patients.

While selection bias cannot be excluded, our results show that gender should not be a reason for withholding VT ablation.

### Limitations

Our present study is limited by its observational design, the size of the patient population and the single-center design. Our study population is comprised of structural heart disease patients, however, we did not discriminate between those with higher risk of SCD, such as patients with hypertrophic cardiomyopathy or arrhythmogenic right ventricular cardiomyopathy (27, 28).

Similarly, to previous studies, the underrepresentation of females largely impacts the number of female patients who can be included in the analysis. Consequently, the number of women reaching each respective endpoint is relatively small, thus it is a major limitation of this study. The available data has thus insufficient power for multivariate Cox regression; for this reason, we were unable to identify independent predictors in the female population. However, our conclusions are mainly driven by the results detailed above, therefore should be interpreted and addressed accordingly.

In our registry, certain values were incomplete or missing. The ablations were performed in our tertiary referral center; therefore, the observed population might have been subject to referral bias. We also lack data about the cause of death in many patients due to the retrospective design.

The RFA techniques and technologies improved immensely over the last couple of years, which might have influenced the outcomes. Operators always followed the actual European guideline recommendations in terms of ablation indication or procedure technique, however, we are unable to provide a timeline of such changes in our practice, as it is not available in our registry. In addition to that, some technologies (such as MitraClip) were not available in our institute at the time, which also could influence the outcomes.

State-of-the-art measures of diastolic dysfunction were only partially available for our patient population, therefore only E/A ratio and DT could be assessed at this time. A larger study using speckle tracking analysis to estimate diastolic function is planned soon.

## Conclusions

Sex differences are certainly present in case of ventricular arrhythmias. Recurrence predictors in men are dominantly associated with severe heart failure and malignant arrhythmia behavior. Systolic heart failure has the largest influence on 1-year mortality. On the other hand, in women, diabetes is a key mortality predictor after VT ablation. Concomitant treatment with ACEi/ARBs was associated with better outcomes. Due to the limitations detailed above, our results are mostly hypothesis generating. Prospective analyses on a larger patient cohort are necessary to investigate the sex differences of patients undergoing VT ablation.

## Data availability statement

The raw data supporting the conclusions of this article will be made available by the authors, without undue reservation.

## Ethics statement

The studies involving human participants were reviewed and approved by Semmelweis University Scientific and Ethics Committee. Written informed consent for participation was not required for this study in accordance with the national legislation and the institutional requirements.

## Author contributions

All authors listed have made a substantial, direct, and intellectual contribution to the work and approved it for publication.

## Funding

Project no. NVKP_16-1–2016-0017 (National Heart Program) has been implemented with the support provided from the National Research, Development, and Innovation Fund of Hungary, financed under the NVKP_16 funding scheme. The research was financed by the Thematic Excellence Program (2020-4.1.1.-TKP2020) of the Ministry for Innovation and Technology in Hungary, within the framework of the Therapeutic Development and Bioimaging thematic programs of the Semmelweis University.

## Conflict of interest

GS reports personal fees from Boston Scientific, Bayer, Johnson and Johnson Medical and Abbott, not related to the present study. BM receives lecture fees from Biotronik, Medtronic and Abbott. The remaining authors declare that the research was conducted in the absence of any commercial or financial relationships that could be construed as a potential conflict of interest.

## Publisher's note

All claims expressed in this article are solely those of the authors and do not necessarily represent those of their affiliated organizations, or those of the publisher, the editors and the reviewers. Any product that may be evaluated in this article, or claim that may be made by its manufacturer, is not guaranteed or endorsed by the publisher.
